# COPB2 promotes hepatocellular carcinoma progression through regulation of YAP1 nuclear translocation

**DOI:** 10.32604/or.2025.058085

**Published:** 2025-03-19

**Authors:** BIAO WU, XIANLIN GUO, ZHISHI WU, LIANG CHEN, SUQING ZHANG

**Affiliations:** 1 Department of General Surgery, Changhai Hospital, Second (Navy) Military Medical University, Shanghai, 200433, China; 2 Department of General Surgery, Zhengzhou First People’s Hospital, Zhengzhou, 450000, China; 3 Department of Hepatobiliary and Pancreatic Surgery, Affiliated Tumor Hospital of Nantong University, Nantong, 226361, China

**Keywords:** Coatomer protein complex subunit beta 2 (COPB2), Yes-associated protein 1 (YAP1), Hepatocellular carcinoma (HCC) prognosis, Cisplatin (DDP)

## Abstract

**Objectives:**

Although Yes-associated protein 1 (YAP1) is an important oncogene in hepatocellular carcinoma (HCC) progression, its nuclear localization prevents it from being considered a potential therapeutic target. Recently, studies have reported that coatomer protein complex subunit beta 2 (COPB2) also plays a critical role in HCC development; however its mechanism of action is unclear. This study aimed to investigate the role of COPB2 and YAP1 in the progression of HCC and to elucidate the underlying mechanisms.

**Methods:**

COPB2 and YAP1 expression in HCC tissues were first analyzed by database searches and immunohistochemistry. Nomogram and artificial neural network models were established based on COPB2 and YAP1 expression. Cell proliferation was detected by cell counting kit-8 and clone formation assay, while cell migration and invasion were assessed using Transwell assays. Finally, the potential mechanisms underlying COPB2 regulation of YAP1 nuclear translocation were explored by immunofluorescence assay and Western blot.

**Results:**

COPB2 combined with YAP1 expression was associated with overall postoperative survival in HCC patients and was an independent prognostic factor. High expression of both COPB2 and YAP1 in patients may reduce the efficacy of postoperative transarterial chemoembolization therapy. *In vitro* experiments revealed that COPB2 affected the sensitivity of HCC cells to Cisplatin (DDP) by regulating YAP1 nuclear translocation.

**Conclusions:**

Our findings suggest that COPB2/YAP1 affects the drug sensitivity of HCC cells to DDP and that targeting COPB2/YAP1 may be a promising strategy for the precision treatment of HCC.

## Introduction

Hepatocellular carcinoma (HCC) is the sixth most common cancer and was the third leading cause of cancer death worldwide in 2020 [1]. Most HCC patients in China have a history of hepatitis B virus infection and the resulting cirrhosis. Many patients are already in the middle to late stages at diagnosis and therefore have a poor prognosis. The 5-year survival rate for advanced HCC patients is less than 5% [2]. In addition to liver transplantation, radical hepatectomy and ablation are the most effective treatments for early-stage HCC patients [3]. Transarterial chemoembolization (TACE) is the first-line treatment for patients with intermediate to advanced HCC in the United States, Asia, and Europe [4]. It is indicated for unresectable patients who have relapsed after surgery or have a limited disease and satisfactory preserved liver function [5]. Despite increasing improvements in the management and perioperative care of HCC patients, recurrence and metastasis remain key challenges to long-term survival [6]. Therefore, the search for mining novel biomarkers is crucial for improving patient prognosis [7–9]. In addition, the study of HCC-related genes and molecular biological mechanisms is of great clinical significance in predicting the prognosis of HCC patients and identifying potential targets for HCC therapy.

The Hippo signaling pathway is a significant cancer suppression pathway and plays a crucial role in inhibiting the proliferation and survival of HCC cells [10] The Hippo pathway was initially identified and named by screening for mutant tumor suppressors in Drosophila [11]. The main components of the pathway include the serine-threonine kinase module and the transcriptional module [12]. The Hippo pathway has significant regulatory functions for organ development, regeneration, and stem cell biology [13]. Once activated, the Hippo pathway undergoes a series of kinase phosphorylation reactions that ultimately lead to the inability of the phosphorylated downstream transcription factor Yes-associated protein 1 (YAP1) to enter the nucleus to activate gene transcription [14]. In contrast, inactivation of the Hippo pathway in tumors results in YAP1 dephosphorylation, and entry of dephosphorylated YAP1 into the nucleus induces cell proliferation and expression of anti-apoptotic genes [15]. There is growing evidence that YAP1 is an essential oncogene and a downstream target of the Hippo pathway [16]. Intranuclear YAP1 expression is vital role in predicting postoperative survival and tumor recurrence in patients with HCC [17]. In addition, YAP1 has prognostic implications for patients treated with TACE. Although the core components of the Hippo pathway have been studied in detail, the upstream regulators remain unclear.

The coatomer protein complex subunit beta 2 (COPB2) is a component subunit of the coatomer complex I (COPI), a nuclear protein containing 906 amino acid residues and with a molecular weight of 102 kD [18]. COPB2 has a tryptophan-aspartate (WD) repeat sequence, which is associated with signal transduction, regulation of the cell cycle and apoptosis [19]. COPB2 interacts with ADP-ribosylation factor, GTPase-activating protein 2, and KKXX-containing substances, and the products of these reactions are COPI-coated vesicles, which play a crucial role in vesicle transport and Golgi budding between the endoplasmic reticulum and Golgi apparatus [20]. Wang et al. and Couzens et al. found that vesicle transport regulates many signaling pathways, including the Hippo pathway, through proteomic studies [21]. Vesicular transport can affect the stability of YAP1 by regulating its phosphorylation [22]. In addition, COPB2 is abnormally highly expressed in many tumor tissues, including colon cancer [23], prostate cancer [24], and gallbladder cancer [25]. An et al. reported that COPB2 inhibits the progression of gastric cancer through the receptor tyrosine kinase pathway [26]. Pu et al. also found that COPB2 promotes lung cancer progression by regulating YAP1 nuclear translocation [27]. In a recent study, COPB2 promoted HCC progression by regulating the epithelial-mesenchymal transition pathway [28]. These results indicate that COPB2 plays a significant role in cancer development. However, the correlation between COPB2 and YAP1 in HCC has not been investigated.

Our study found that COPB2 combined with YAP1 predicted the prognosis of HCC patients and developed associated nomogram and artificial neural network (ANN) prediction models. In addition, we found that COPB2 reduced the drug sensitivity of HCC cells to Cisplatin (DDP) by regulating YAP1 nuclear translocation. This finding may be critical in the progression and metastasis of HCC. Therefore, understanding this pathway may help identify new methods for treating HCC.

## Materials and Methods

### Database analysis

The Human Protein Atlas web (http://www.proteinatlas.org/) (accessed on 12 December 2024) was used to obtain relevant immunohistochemical staining images of COPB2 and YAP1 in HCC tissues.

### Patient and clinical samples

We retrospectively analyzed two independent cohorts comprising 214 HCC patients. The training group randomly selected 114 HCC patients from the Affiliated Hospital of Nantong University between 2012 and 2017. Moreover, 100 HCC patients from the Affiliated Tumor Hospital of Nantong University between 2011 and 2016 were randomly selected as the validation group. Cases were selected according to the following criteria: no anti-cancer-related treatment before surgery, the pathological diagnosis of HCC, and availability of resected tissue and follow-up data. Patients underwent physical examination, laboratory diagnosis, and imaging twice a year after surgery. Most patients underwent TACE after relapse. Overall survival (OS) was measured from the date of surgery to the date of death or last follow-up. For each patient, the following clinicopathological information was collected: age, sex, hepatitis B virus, presence of cirrhosis, serum alpha-fetoprotein level, tumor number, tumor size, degree of tumor differentiation, vascular tumor thrombus, and lymph node metastasis. The staging of tumors was defined according to the American Joint Committee on Cancer/International Union Against Cancer Tumor-Lymph Node Metastasis (TNM) classification system. Written informed consent was obtained from each patient included in the study. The study protocol conforms to the ethical guidelines of the 1975 Declaration of Helsinki, as reflected in a prior approval by the institution’s human research committee.

### Immunohistochemistry

Tissue microarray (TMA) sections were produced by the pathology departments of both hospitals. They were placed on a paraffin section spreader and baked for 20 min. After drying, TMA were successively immersed in xylene I and II for 15 min each, and then in anhydrous ethanol, 95% and 75% ethanol for 5 min each. The sections were washed three times with PBS water for 5 min each. The endogenous peroxidase was then blocked from inactivation by immersion in 3% H_2_O_2_ solution for 10 min. After 10 min it was washed 3 times with PBS. Water was first boiled in an autoclave and prepared EDTA alkaline restorative solution was added to a porcelain jar. Place the slide on a metal staining rack into the porcelain cylinder and then place it in the autoclave, cover with a stainless steel lid and pressurise slowly, when the small valve rises, continue to heat for 3 min and then remove the heat source, place the autoclave in cool water and open the lid when the small valve sinks. Cool naturally to room temperature and rinse with PBS. Next, the sections were incubated with rabbit polyclonal anti-COPB2 antibody (ab229639, 1:1000, Abcam, Cambridge, UK) and YAP1 monoclonal antibody (ab205270, 1:200, Abcam, Cambridge, UK) overnight at 4°C After washing three times with PBS, the sections were incubated with secondary antibodies (ab288151 1:4000, Abcam, Cambridge, UK) for 20 min at room temperature, and then diaminobenzidine solution (D8001, Merck, Shanghai, China) was applied. Finally, sections were re-stained with hematoxylin, dehydrated, and fixed. All microarrays were scored independently by two experienced pathologists. COPB2 scoring criteria: The percentage of positive cells was evaluated on a scale of 0–3: 0 (0%–25%), 1 (26%–50%), 2 (51%–75%), and 3 (76%–100%), and classified into low (0–1 point) and high (2–3 points) expression groups. YAP1 scoring criteria: A percentage of positive nuclei greater than 10% was recorded as high expression and less than 10% was recorded as low expression.

### Western blot

HCC cells were lysed on ice in a cell lysis buffer (P0013J, Beyotime, Shanghai, China). The protein concentration was determined using the bicinchoninic acid assay (P0012, Beyotime, Shanghai, China). Electrophoresis was then performed on sodium dodecyl sulfate-polyacrylamide gel electrophoresis gels and transferred to polyvinylidene difluoride (PVDF) membranes (88585, Thermo Fisher Scientific, Franklin, MA, USA). Transfer the PVDF membrane to 4°C containment buffer (37538, Gibco, Grand 93 Island, NY, USA) and block for 2 h at room temperature. Incubate the diluted antibody with the membrane at 4°C overnight. After removing the membrane the next day it was left at room temperature for 1 h. Incubate the secondary antibody (1:5000, 31430, 31460, Gibco) for 2 h at room temperature. The signals were detected by an ECL system (Amersham Pharmacia, Piscataway, NJ, USA). The following antibodies were used in this study: COPB2 (ab229639, 1:1000, rabbit, Abcam, Cambridge, UK), YAP1 (ab205270, 1:500, rabbit, Abcam, Cambridge, UK), pLATS1 (8654T, 1:500, rabbit, Cell Signaling Technology, Danvers, MA, USA), pYAP1 (49115, 1:500, rabbit, Cell Signaling Technology, Massachusetts, USA), and GAPDH (ab8245, 1:5000, mouse, Abcam, Cambridge, UK). In order to ensure the reliability and reproducibility of the experiments: All experiments were repeated 3 times.

### Cell culture and vectors

The human HCC cell lines Huh7 and SK-hep1 were purchased from the Academy of Sciences Committee of the China Cell Resource Centre (Shanghai, China). The cells were cultured in Dulbecco’s modified Eagle 92 medium (11965092, DMEM, Hyclone, Logan, UT, USA) containing 10% fetal bovine serum (A5670401, FBS, Gibco) and 1% antibiotic-antimycotic (15240062, Gibco) at 37°C in a humidified atmosphere of 5% CO_2_. HCC cells in the exponential growth phase were used for subsequent experiments. No mycoplasma contamination was found in all cells. The COPB2 siRNA was designed by Polyplus (Sense: CCCAUUAUGUUAUGCAGAUTT; Antisense–1: AUCUGCAUAACAUAAUGGGTT). YAP1/pcDNA3.1 was constructed, and the sequence was examined using Genechem (Shanghai, China). For transient expression, plasmids were transfected with lipofectamine 2000 (11668030, Invitrogen, Shanghai, China) for 24 h, after which the cells were processed with the indicated reagents described above. In order to ensure the reliability and reproducibility of the experiments: at least 3 replicate wells are set up within the group in the transfection experiments; the same amount of cell inoculum is guaranteed, and the cells are evenly distributed.

### Cell counting kit-8 (CCK-8) assay

The Huh7 and SK-hep1 cells were digested with 0.25% trypsin and diluted to a 5 × 10^4^ cells/mL concentration. The cells were counted using the Countess™ 3 Automated Cell Counter (Thermo Fisher Scientific, Franklin, MA, USA), and the cell suspension was inoculated into 96-well plates at a density of 2000 cells/well. CCK-8 solution (10 μL) (C6050, NCM, Suzhou, China) was added to each well and cultured at 37°C for 2 h. Cell viability was determined by measuring the absorbance at 450 nm. In order to ensure the reliability and reproducibility of the experiments: Both normal and control groups were set up with 8 replicate holes and all experiments were repeated 3 times.

### Clone formation assay

Briefly, Huh7 and SK-hep1 cells (2000 cells/well) were inoculated in 6-well plates and incubated at 37°C. The medium was changed every three days, and cell growth was observed. Fourteen days after inoculating cells, colonies with a cell count >50 were observed under a microscope (DM8000 M, Leica, Mannheim, Germany). Cells were washed with PBS and fixed in 4% paraformaldehyde (BL539A, Biosharp, Hefei, China) for 20 min at room temperature. The fixed cells were stained with 1 mL of crystal violet staining solution (C0121, Beyotime, Shanghai, China) for 10 min, washed with PBS, and air-dried at room temperature. The cells were photographed, and the visible clones were counted. In order to ensure the reliability and reproducibility of the experiments: Both normal and control groups were set up with 3 replicate holes and all experiments were repeated 3 times.

### Transwell assay

Migration assay: Transwell chambers were placed in 24-well plates; 2 × 10^5^ Huh7 and SK-hep1 cells starved in 200 μL of serum-free medium were added to the upper chamber, and 500 μL of medium with 10% serum was added to the lower chamber. After 24 h of incubation at 37°C and 5% CO_2_, the Transwell chambers were first fixed in paraformaldehyde for 30 min and then stained with crystal violet staining solution for 10 min. The cells were photographed and counted under the microscope, with three replicates per group.

Invasion assay: Matrigel (354248, Corning, NY, USA) was melted at 4°C overnight and diluted with pre-cooled serum-free DMEM medium. Next, 100 µL of diluted Matrigel was added to the upper chamber. The remaining steps are the same as those in the Transwell migration assay. In order to ensure the reliability and reproducibility of the experiments: Both normal and control groups were set up with 3 replicate holes and all experiments were repeated 3 times.

### Immunofluorescence

Huh7 and SK-hep1 cells were inoculated in 24-well plates for 24 h. The cells were then fixed in 4% paraformaldehyde for 15 min and permeabilized for 10 min. After being closed with 5% Bovine Serum Albumin (BSA, 9998, Cell Signaling Technology, Danvers, MA, USA) for 1 h, the cells were incubated overnight at 4°C with YAP1 antibody (ab205270, 1:100, Abcam, Cambridge, UK) and then Alexa Fluor 594 anti-rabbit IgG secondary antibody (A21441, Invitrogen, Shanghai, China). Furthermore, the cell nuclei were stained with DAPI (D1306, Thermo Fisher Scientific, Massachusetts, USA), and photographs were taken under a fluorescent microscope (Leica, Mannheim, Germany). In order to ensure the reliability and reproducibility of the experiments: Both normal and control groups were set up with 3 replicate holes and all experiments were repeated 3 times.

### Statistical analysis

Differences in expression were analysed by paired *t*-test. Clinicopathological characteristics were assessed using the chi-square test. *p* < 0.05 was considered a statistically significant difference. Statistical analyses were performed using the SPSS 26.0 statistical package (SPSS, Inc., Chicago, IL, USA) and GraphPad Prism version 7.01 (GraphPad Software, Inc, La Jolla, CA, USA). Nomogram was developed using the Regression Modelling Strategies package in R (version 3.0.2; R Package “rms” for nomogram establishment; www.r-project.org (accessed on 12 December 2024)). ANN was built using SPSS Clementine version 12.0 software for Windows (IBM Corporation, Shanghai, China).

## Results

### Expression of COPB2 and YAP1 in HCC tissues

**Figure 1:**
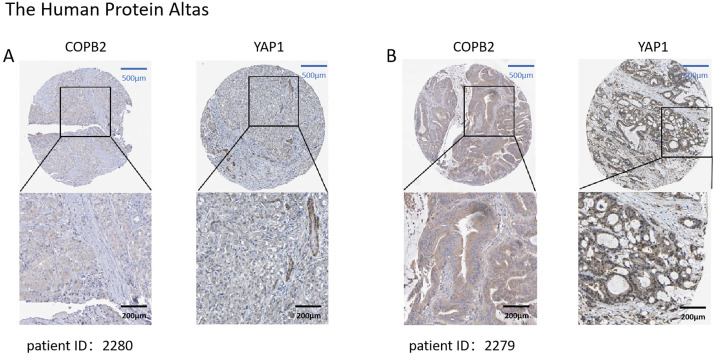
Expression of coatomer protein complex subunit beta 2 (COPB2) and Yes-associated protein 1 (YAP1) in hepatocellular carcinoma (HCC) tissues in the database. (A) In patient 2280, COPB2 expression was low in the cytoplasm, and YAP1 expression was low in the nucleus. (B) In patient 2279, COPB2 was highly expressed in the cytoplasm, while YAP1 was highly expressed in the nucleus.

**Figure 2:**
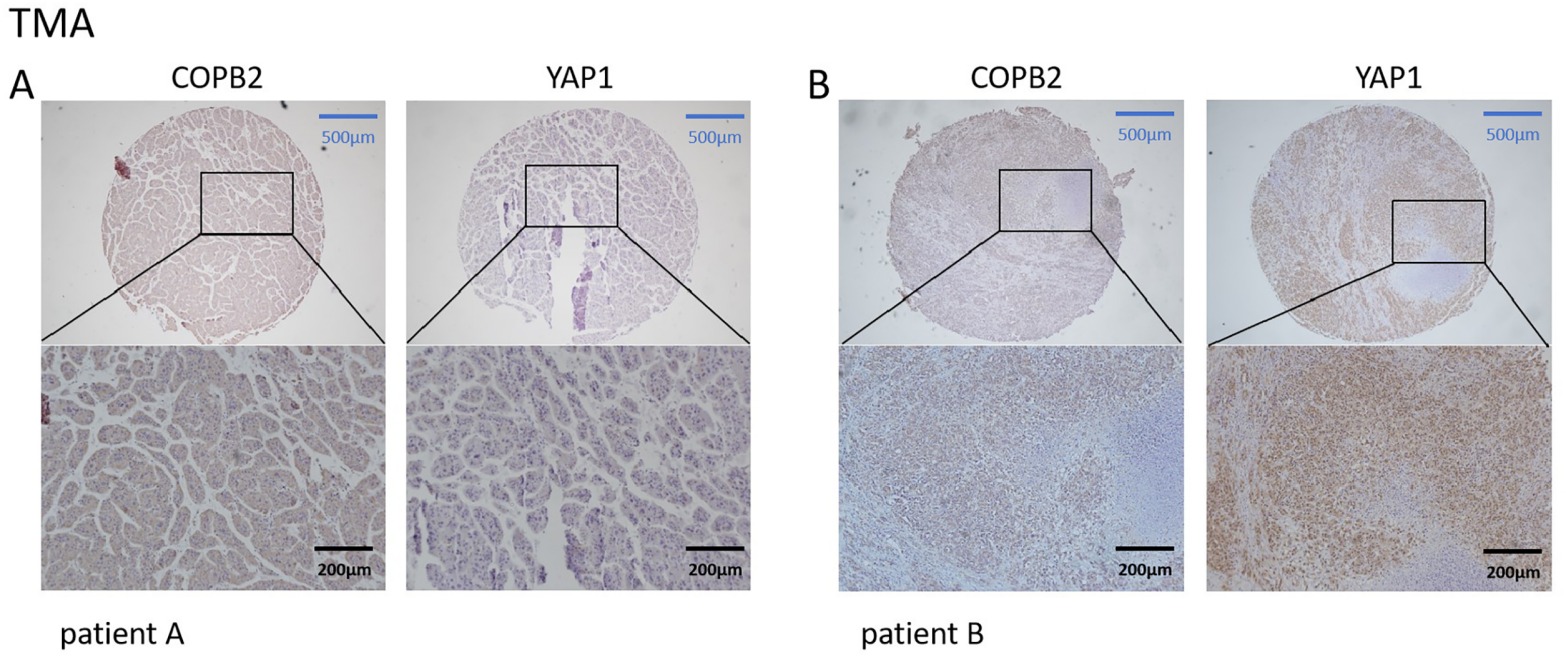
Correlation of coatomer protein complex subunit beta 2 (COPB2) and Yes-associated protein 1 (YAP1) expression in hepatocellular carcinoma tissues in tissue microarrays. (A) In patient A, COPB2 expression was low in the cytoplasm, and YAP1 expression was low in the nucleus. (B) In patient B, COPB2 was highly expressed in the cytoplasm, and YAP1 was highly expressed in the nucleus.

We first searched the database to explore the connection between COPB2 and YAP1 expression in HCC tissues. Notably, when COPB2 was highly expressed in the cytoplasm, YAP1 was also highly expressed in the nucleus. When the COPB2 expression was low, the YAP1 expression also reduced (Fig. 1). It is noteworthy that COPB2 expression in the cytoplasm was positively associated with YAP1 expression in the nucleus. To verify the findings in the database, we performed immunohistochemical staining of COPB2 and YAP1 on tissue microarrays of 214 specimens from two hospitals (Fig. 2). We found that COPB2 was predominantly present in the cytoplasm and YAP1 was expressed in both the nucleus and cytoplasm, and we grouped samples into high and low expression groups based on the two antibody staining scores. Among the 114 HCC specimens in the training group, the COPB2 low and high expression rates were 43.9% (50/114) and 56.1% (64/114), respectively, while the YAP1 low and high expression rates were 42.1% (48/114) and 57.9% (66/114), respectively. Similarly, among the 100 HCC specimens in the validation group, the low and high COPB2 expression rates were 43% (43/100) and 57% (57/100), respectively, and the low and high YAP1 expression rates were 33% (33/100) and 67% (67/100), respectively.

### Correlation of COPB2 and YAP1 expression with the clinicopathological characteristics of HCC patients

As shown in Table 1, higher COPB2 expression was associated with larger tumor diameter and higher staging in both the training and validation groups (*p* < 0.05). Higher YAP1 expression was associated with poorer tumor differentiation and higher staging in both groups (*p* < 0.05) (Table 2).

**Table 1:** Relationship between COPB2 expression and clinicopathological characteristics of HCC

Clinical pathological indexes	Training cohort			Validation cohort		
	(114 cases)			(100 cases)		
	Low	High	*p*	Low	High	*p*
Age (years)			0.72			0.059
<55	22	27		19	36	
≥55	27	38		24	21	
Sex			0.283			0.697
Female	8	16		7	11	
Male	41	49		36	46	
HBV			0.152			0.907
Absent	19	34		11	14	
Present	30	31		32	43	
Cirrhosis			0.242			0.703
Absent	18	31		28	35	
Present	31	34		15	22	
Tumour size (cm)			**0.002**			**0.002**
<5	39	33		34	28	
≥5	10	32		9	29	
Serum Alpha-Fetal Protein (AFP ng/mL)			0.751			0.146
<400	38	52		33	36	
≥400	11	13		10	21	
Tumour number			0.883			0.587
1	39	51		33	41	
≥2	10	14		10	16	
Differentiation			0.061			**0.036**
Well	5	15		18	14	
Moderate	26	37		18	21	
Poor	18	13		7	22	
Vascular tumour thrombus			0.052			0.133
Absent	41	44		37	42	
Present	8	21		6	15	
Regional lymph node metastasis			0.153			0.179
Absent	40	59		40	48	
Present	9	6		3	9	
TNM stage			**<0.001**			**0.001**
I + II	36	25		33	25	
III + IV	13	40		10	32	

**Table 2:** Relationship between YAP1 expression and clinicopathological characteristics of HCC

Clinical pathological indexes	Training cohort			Validation cohort		
	(114 cases)			(100 cases)		
	Low	High	*p*	Low	High	*p*
Age (years)			0.279			**0.002**
<55	28	45		11	44	
≥55	20	21		22	23	
Sex			0.607			0.974
Female	9	15		6	12	
Male	39	51		27	55	
HBV			0.904			0.066
Absent	22	31		12	13	
Present	26	35		21	54	
Cirrhosis			0.183			0.594
Absent	20	29		22	41	
Present	28	33		11	26	
Tumour size (cm)			0.291			**0.047**
<5	33	39		25	37	
≥5	15	27		8	30	
Serum AFP (ng/mL)			0.961			0.305
<400	38	52		25	44	
≥400	10	14		8	23	
Tumour number			0.327			0.444
1	40	50		26	48	
≥2	8	16		7	19	
Differentiation			**0.021**			**0.009**
Well	13	7		17	15	
Moderate	27	36		11	28	
Poor	8	23		5	24	
Vascular tumour thrombus			0.067			0.314
Absent	40	45		28	51	
Present	8	21		5	16	
Regional lymph node metastasis			0.345			0.53
Absent	40	59		30	58	
Present	8	7		3	9	
TNM stage			**0.005**			**0.012**
I + II	33	28		25	33	
III + IV	15	38		8	34	

### Correlation of COPB2 and YAP1 expression with the OS of HCC patients

**Figure 3:**
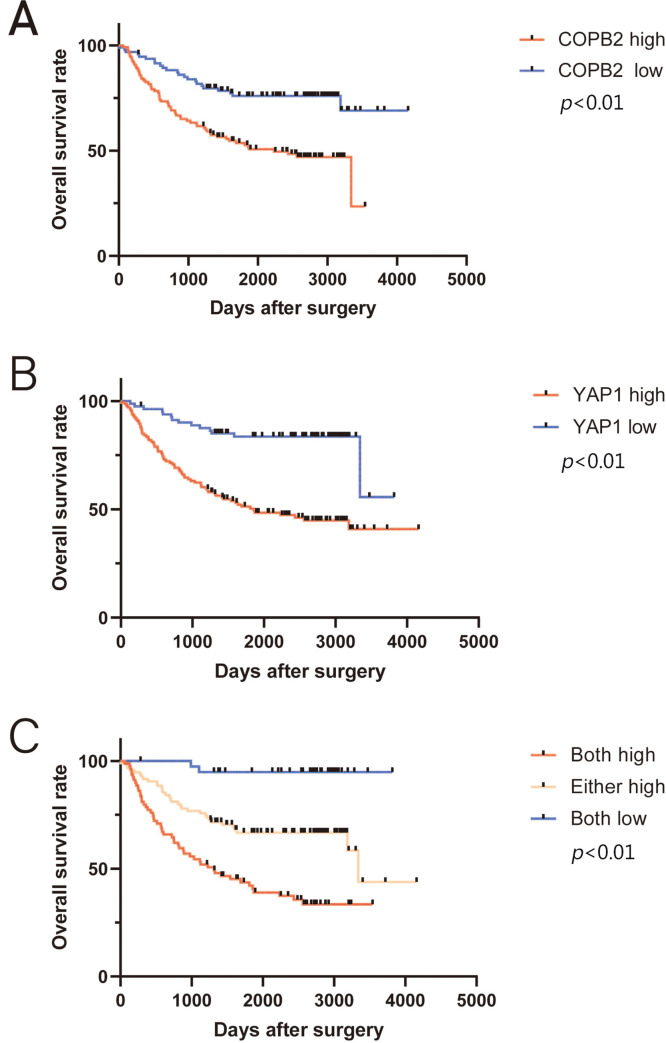
Kaplan-Meier survival analysis between coatomer protein complex subunit beta 2/Yes-associated protein 1 (COPB2/YAP1) expression and overall survival (OS) of hepatocellular carcinoma patients after surgery. (A) Patients with higher COPB2 expression have a shorter OS. (B) Patients with higher YAP1 expression have a shorter OS. (C) Patients with increased expression of both COPB2 and YAP1 have the shortest OS.

We found that the combined COPB2 and YAP1 expression was an independent prognostic factor for HCC patients and the TNM staging in the training and validation groups, by univariate and multivariate analyses, respectively (Table 3). Kaplan-Meier survival analysis showed that patients with lower COPB2 or YAP1 expression had a longer OS (Fig. 3A and B). Moreover, we found that patients with low expression of both COPB2 and YAP1 had longer OS than those with high COPB2 and YAP1expression (Fig. 3C).

**Table 3:** Univariate and multivariate analysis of prognostic factors and OS in HCC patients undergoing surgery

Variable	Training cohort (n = 114)				Validation cohort (n = 100)			
	Univariate		Multivariate		Univariate		Multivariate	
	HR (95% CI)	*p*	HR (95% CI)	*p*	HR (95% CI)	*p*	HR (95% CI)	*p*
COPB2/YAP1 expression (Both high *vs*. Either high *vs*. Both low)	3.096 (1.881–5.096)	**<0.001**	0.074 (0.010–0.561)	**0.012**	3.392 (1.936–5.944)	**<0.001**	0.488 (0.248–0.962)	**0.038**
Age (<55 years *vs*. ≥55 years)	0.687 (0.380–1.242)	0.557			1.632 (0.863–3.087)	0.132		
Sex ( male *vs*. female)	1.296 (0.654–2.565)	0.457			0.754 (0.360–1.581)	0.455		
HBV (Absent *vs*. Present)	0.670 (0.370–1.214)	0.186			0.962 (0.469–1.971)	0.915		
Cirrhosis (Absent *vs*. Present)	0.977 (0.538–1.775)	0.94			0.954 (0.500–1.821)	0.887		
Tumour size (<5 cm *vs*. ≥5 cm)	1.316 (0.721–2.401)	0.371			0.296 (0.158–0.555)	**<0.001**		
Serum AFP (<400 ng/mL *vs*. ≥400 ng/mL)	0.538 (0.227–1.274)	0.159			1.483 (0.784–2.805)	0.225		
Tumour number (1 *vs*. ≥2)	1.600 (0.823–3.108)	0.166			1.616 (0.846–3.087)	0.146		
Differentiation (Well *vs*. Moderate and Poor)	0.468 (0.296–0.741)	**0.001**			0.164 (0.061–0.441)	**<0.001**		
Vascular tumour thrombus (Absent *vs*. Present)	2.310 (1.256–4.250)	**0.007**			0.442 (0.228–0.857)	**0.016**		
Regional lymph node metastasis (Absent *vs*. Present)	1.225 (0.546–2.748)	0.623			2.039 (0.902–4.609)	0.087		
TNM stage (I + II *vs*. III + IV)	4.040 (2.107–7.745)	**<0.001**	0.362 (0.181–0.724)	**0.004**	0.233 (0.120–0.451)	**<0.001**	0.287 (0.147–0.558)	**<0.001**

### Nomogram and ANN model based on COPB2 and YAP1 expression

**Figure 4:**
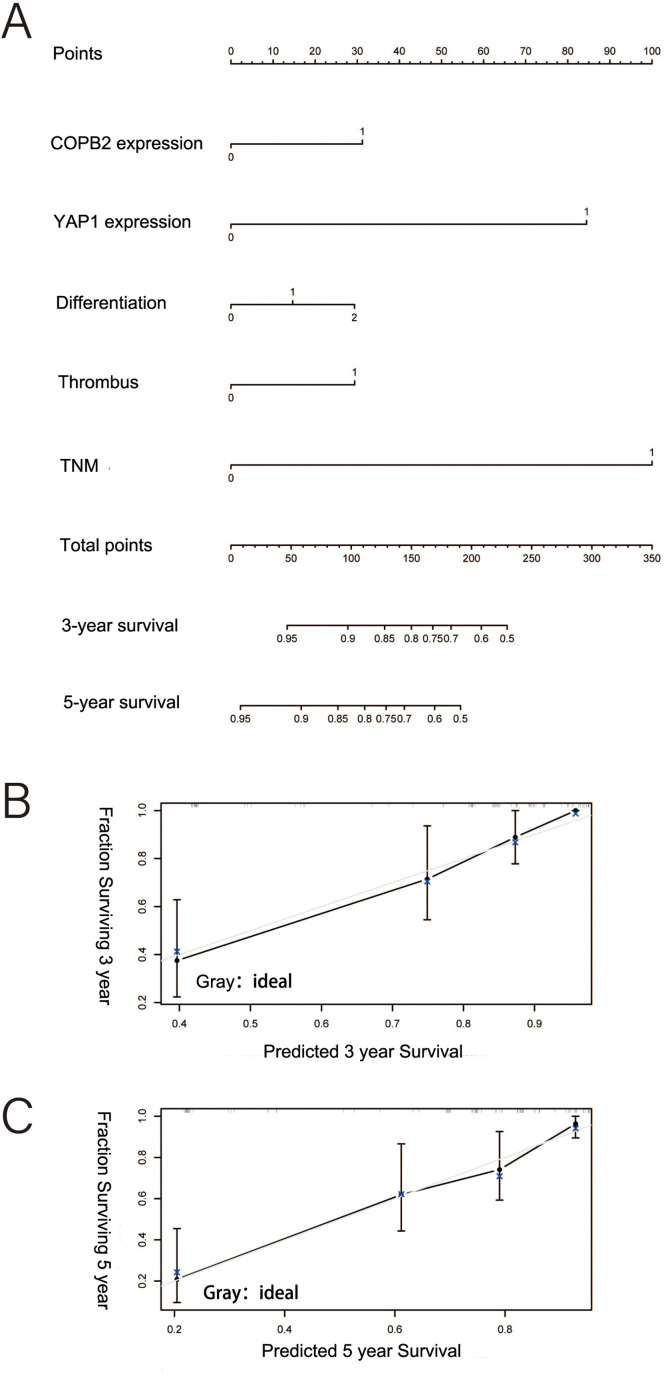
Nomogram predicting the probability of survival at 3 and 5 years. (A) Nomogram of hepatocellular carcinoma patients after surgery based on coatomer protein complex subunit beta 2 and Yes-associated protein 1 expression. (B–C) Good calibration for predicting survival at 3 and 5 years.

Based on the nomogram, we scored several important clinicopathological features for each patient, and the sum of the points obtained was used to predict the 3- and 5-year survival rates of HCC patients (Fig. 4A). A higher score predicts a worse prognosis for the patient. The model showed good accuracy in predicting the OS of HCC patients after hepatectomy with a c-index of 0.673. Calibration plots show the model’s good prediction of 3- and 5-year survival rates for HCC patients (Fig. 4B and C).

**Figure 5:**
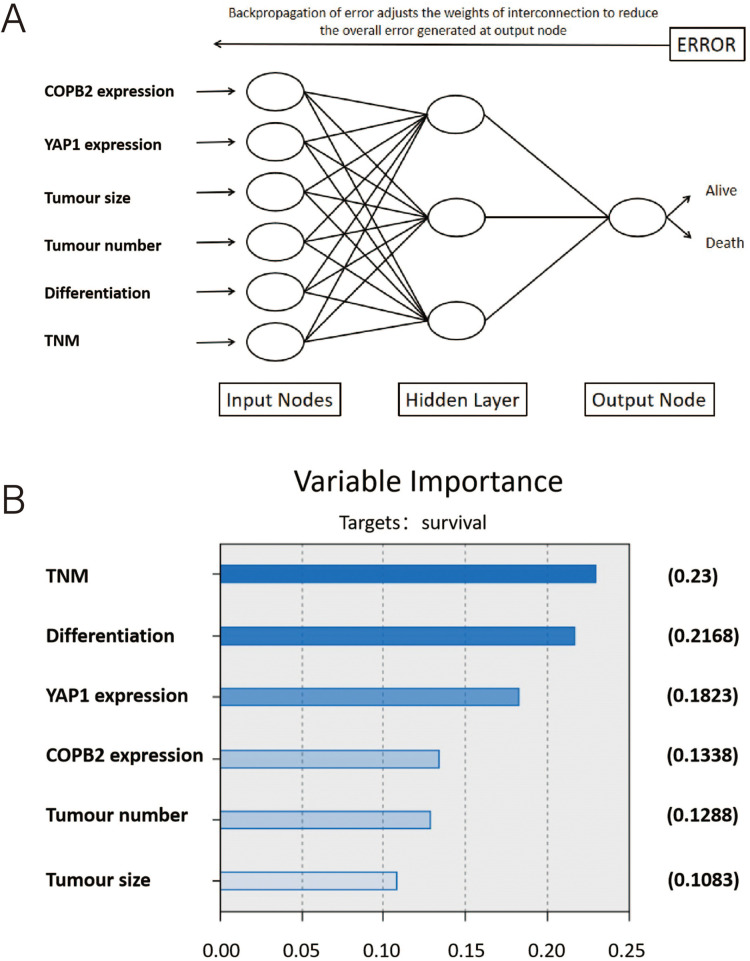
An ANN model related to COPB2 and YAP1 expression. (A) Schematic representation of an artificial neural network (ANN) for predicting overall survival after surgery in patients with hepatocellular carcinoma. (B) The importance of the variables in the ANN model.

Similarly, an ANN model related to COPB2 and YAP1 expression was developed based on several important risk factors (Fig. 5A). The proportion of OS importance accounted for by risk factors of TNM staging, degree of tumor differentiation, YAP1 expression, COPB2 expression, tumor number, and tumor size in the ANN model was 0.23, 0.2168, 0.1823, 0.1338, 0.1288, and 0.1083, respectively (Fig. 5B).

### Correlation between COPB2 and YAP1 expression and OS in HCC patients treated with TACE after surgery

**Figure 6:**
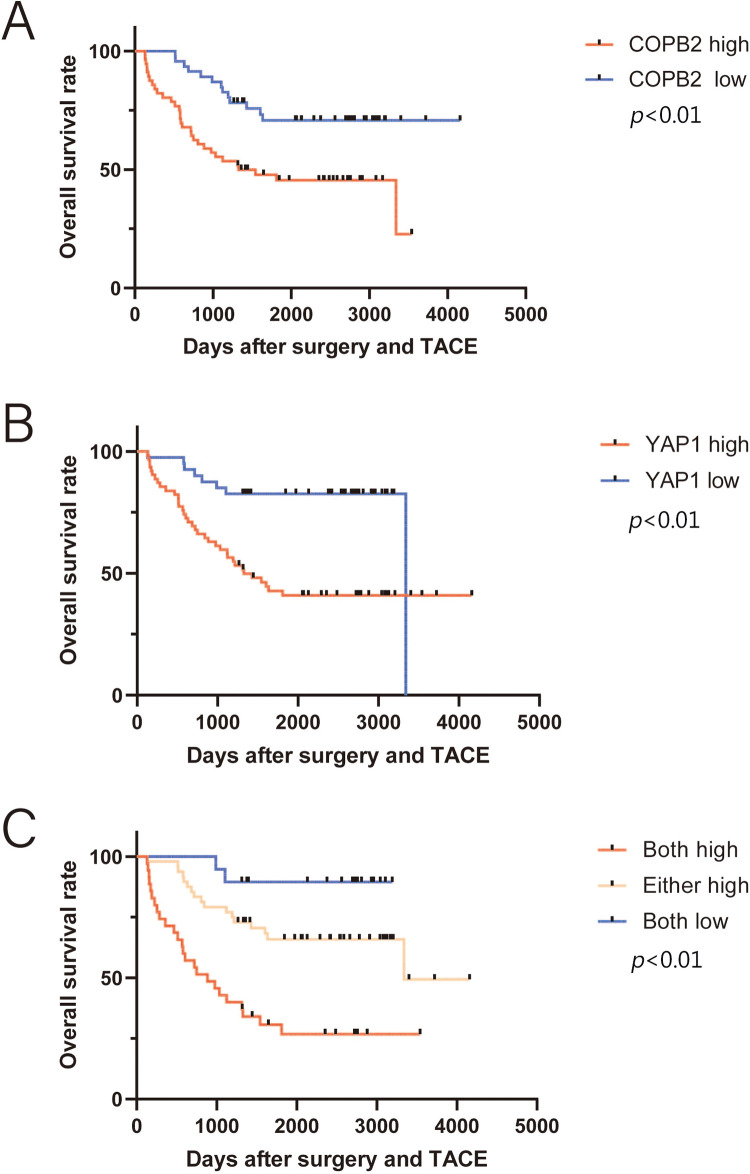
Kaplan-Meier survival analysis between coatomer protein complex subunit beta 2/Yes-associated protein 1 (COPB2/YAP1) expression and overall survival (OS) of hepatocellular carcinoma patients receiving transarterial chemoembolization after surgery. (A) Patients with higher COPB2 expression have a shorter OS. (B) Patients with higher YAP1 expression have a shorter OS. (C) Patients with increased expression of both COPB2 and YAP1 have the shortest OS.

TACE is the first-line treatment for intermediate to advanced HCC. Of the 214 patients at both hospitals, 102 underwent postoperative TACE, and DDP was used in TACE at both hospitals. Kaplan-Meier survival analysis showed that among these patients, those with lower COPB2 and YAP1 expression had a longer OS (Fig. 6). Multivariate analysis identified COPB2 combined with YAP1 expression, vascular tumor thrombus, and TNM staging as independent risk factors (Table 4).

**Table 4:** Univariate and multivariate analysis of prognostic factors and OS in HCC patients undergoing postoperative TACE

	Univariate		Multivariate	
	HR (95% CI)	*p*	HR (95% CI)	*p*
COPB2/YAP1 expression (both high *vs*. either high *vs*. both low)	0.046 (0.006–0.337)	**0.002**	0.495 (0.263–0.931)	**0.029**
Age (<60 years *vs*. ≥60 years)	0.704 (0.387–1.280)	0.25		
Sex ( male *vs*. female)	1.265 (0.889–1.800)	0.192		
HBV (Absent *vs*. Present)	0.941 (0.497–1.782)	0.853		
Cirrhosis (Absent *vs*. Present)	1.178 (0.651–2.132)	0.589		
Tumour size (<5 cm *vs*. ≥5 cm)	1.607 (0.889–2.907)	0.116		
Serum AFP (<400 ng/mL *vs*. ≥400 ng/mL)	1.229 (0.641–2.357)	0.534		
Tumour number (1 *vs*. ≥2)	0.467 (0.250–0.875)	**0.017**		
Differentiation (Well *vs*. Moderate and Poor)	1.197 (0.787–1.820)	0.401		
Vascular tumour thrombus (Absent *vs*. Present)	0.346 (0.188–0.636)	**0.001**	0.506 (0.271–0.943)	**0.032**
Regional lymph node metastasis (Absent *vs*. Present)	0.918 (0.388–2.173)	0.845		
TNM stage (I + II *vs*. III + IV)	0.300 (0.159–0.567)	**<0.001**	0.482 (0.248–0.937)	**0.031**

### COPB2 mediates the drug sensitivity of HCC cells to DDP through the regulation of YAP1

**Figure 7:**
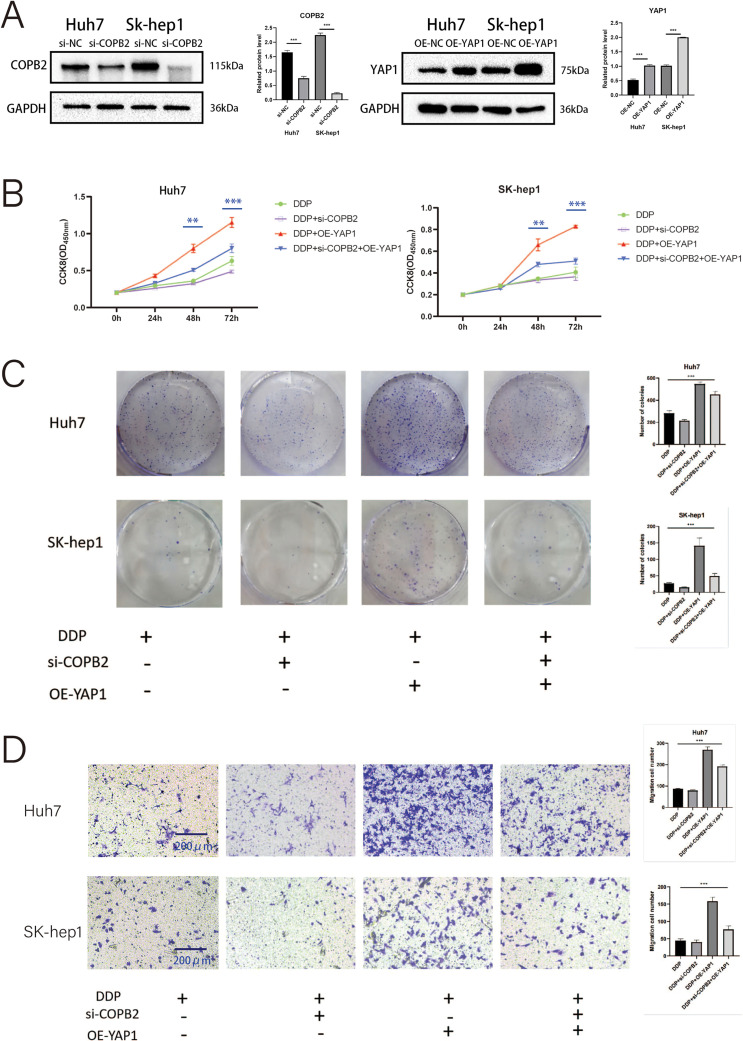

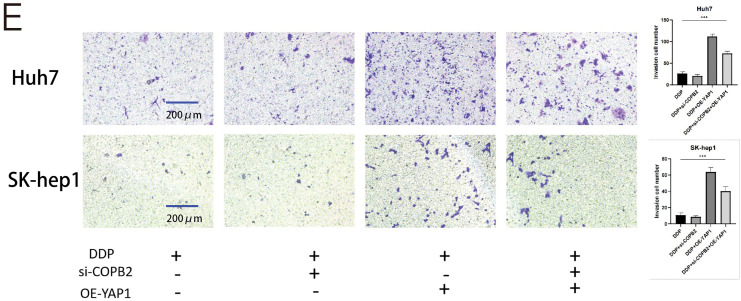
Coatomer protein complex subunit beta 2 (COPB2) mediates the drug sensitivity of hepatocellular carcinoma cells to DDP (5 μg/mL) through the regulation of Yes-associated protein 1 (YAP1). (A) Huh7 and SK-hep1 were transfected with si-RNA to knock down COPB2 and plasmids to overexpress YAP1, and Western blot was used to detect the efficiency. (B) Cell proliferation was detected by CCK-8 assay at different time points. (C) Representative picture and quantification of colony formation. (D–E) Representative picture and quantification of the Transwell assay. All data are displayed as mean ± standard deviation (SD). **p < 0.01, ***p < 0.001.

To demonstrate whether COPB2 and YAP1 affect the sensitivity of HCC cells to DDP, we first selected two cell lines, Huh7 and SK-hep1. We used siRNA to knock down COPB2, plasmids to overexpress YAP1 in cells, and Western blot to verify the efficiency of knockdown and overexpression (Fig. 7A). In the presence of DDP, through CCK8 and clone formation analysis, we discovered that knockdown of COPB2 was able to reduce the proliferation ability of HCC cells, while overexpression of YAP1 could reverse this phenomenon (Fig. 7B and C). Furthermore, we found that knockdown of COPB2 inhibited the migration and invasion of HCC cells by the Transwell assay, while overexpression of YAP1 restored the inhibitory effect of COPB2 knockdown (Fig. 7D and E).

### Knockdown of COPB2 promotes YAP1 exit from the nucleus and affects its stability

**Figure 8:**
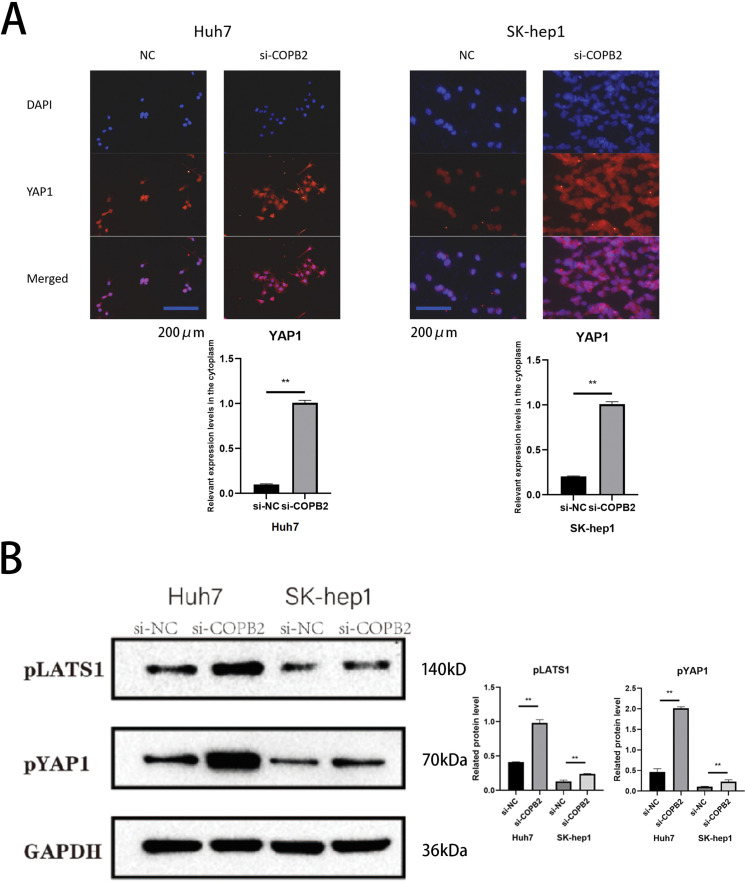
Knockdown of coatomer protein complex subunit beta 2 (COPB2) promotes the exit of Yes-associated protein 1 (YAP1) from the nucleus and affects its stability. (A) Representative images of immunofluorescent staining for YAP1 distribution. (B) Representative pictures of Western blotting analysis of pLATS1 and pYAP1 in Huh7 and SK-hep1 cells transfected with si-NC and si-COPB2. All data are displayed as mean ± standard deviation (SD). **p < 0.01.

Ultimately, by immunofluorescence staining assays, we found that knockdown of COPB2 in Huh7 and SK-hep1 cells promote YAP1 exit from the nucleus (Fig. 8A). In addition, we found increased expression of pLATS1 and pYAP1 after the knockdown of COPB2 (Fig. 8B).

## Discussion

HCC is one of the leading causes of cancer deaths worldwide and remains a major clinical challenge [29]. Despite increasingly sophisticated treatment modalities for HCC patients, the prognosis is still not promising [30]. Therefore, elucidating the molecular mechanisms underlying the pathogenesis of HCC and chemotherapy resistance and exploring potential biomarkers are essential to identify new targeted therapies and improve the prognosis of HCC patients. COPB2 is responsible for the development of many human cancers [31], such as cutaneous squamous cell carcinoma [32], and breast cancer [33]. However, studies on COPB2 in HCC are fewer and more limited. In this study, we found that COPB2 was associated with the Hippo signaling pathway and YAP1 and correlated with prognosis and drug sensitivity in HCC patients. There is increasing evidence that YAP1 is becoming an appealing target for cancer treatment and contributing to our chemotherapy resistance insights [34]. It is noteworthy that we obtained similar results in this study through TMA, survival analysis, and cellular experiments. In this study, we first obtained immunohistochemical profiles of COPB2 and YAP1 in HCC tissues through database searches. After preliminary analysis, we discovered that the cytoplasmic expression of COPB2 was positively associated with the cytosolic expression of YAP1. To verify this phenomenon, we retrospectively analyzed TMA specimens from HCC patients who underwent surgical treatment at two hospitals and divided them into training and validation groups. By immunohistochemical staining of the TMA, we obtained similar results. By chi-square test, we found that COPB2 expression was significantly associated with tumor size and TNM staging in HCC patients, and YAP1 expression was significantly correlated with the degree of tumor differentiation and TNM staging in HCC patients. This result suggests a correlation between COPB2 and YAP1 expression and the tumor load and staging of the patients. It is well known that the prognosis of HCC patients is strongly influenced by tumor load and TNM staging. To further explore whether a combination of these two genes was associated with OS in HCC patients, we found that both high expression of COPB2 and YAP1 and TNM staging were independent prognostic factors for patients undergoing radical hepatic resection for HCC by univariate and multivariate analyses. Kaplan-Meier survival analysis showed a shorter OS for patients with high expression of both COPB2 and YAP1 than for those with low expression. To further investigate the prognostic value of combined COPB2 and YAP1 expression on the clinical outcome of HCC patients, we generated a nomogram, which predicted 3- or 5-year survival rates for HCC patients. In recent years, ANN technology has been widely used in medical diagnosis and prediction. It is a mathematical model of neural algorithms for large-scale or distributed, parallelizable information processing or behavioral decision-making by establishing a way in which the human brain can completely mimic various animal neural network systems. It can completely rely only on the network system to transmit a large amount of information to achieve the purpose of processing data. It easily realizes the purpose of processing massive information by adjusting the parameters of a large number of information nodes connected within the computer system in the network environment in real time and automatically, the interconnections between the nodes, and the parameters of topological spatial relationship of information between the connected nodes, etc., which is an emerging method of establishing a predictive model of the risk of diseases [35,36]. According to our model, YAP1 and COPB2 expression were second only to TNM staging and degree of differentiation in predicting OS in HCC patients.

Further data mining of HCC patients in TMA revealed that 102 patients had undergone at least one TACE treatment after surgery for tumor recurrence or consolidation, of which DDP was the commonly used drug in TACE. We also performed univariate and multivariate analyses to understand whether double-positive COPB2 and YAP1 expression are prognostic guides for HCC patients undergoing postoperative TACE treatment. Surprisingly, we discovered that COPB2 combined with YAP1 expression was also an independent risk factor for this group of patients, suggesting that they play an essential role in assessing the prognosis of HCC patients. Moreover, Kaplan-Meier survival analysis also indicated that patients with high expression of both COPB2 and YAP1 had the worst prognosis. In colon cancer, YAP activation is associated with DDP resistance. Therefore, we speculate that the high expression of COBP2 and YAP1 may be related to the chemical sensitivity of HCC patients to DDP.

*In vitro* experiments showed that HCC cells were more sensitive to DDP after COPB2 knockdown. However, overexpression of YAP1, in addition to this, partially reversed this phenomenon. YAP1 can regulate the sensitivity of cancer cells to drugs through various mechanisms, such as the HIPPO-YAP1-transcriptional enhancer associate domain (TEAD) signaling pathway, stem cell markers, and increased expression of ABC transporter proteins; however, all these mechanisms must rely on YAP1 entering the nucleus for this to occur [37,38]. Consistent with our speculation, upregulation of YAP1 increased the short- and long-term viability of tumor cells under DDP treatment. Subsequent immunofluorescence experiments revealed a significant increase in YAP1 in the cytoplasm of HCC cells following the knockdown of COPB2. Activation of the Hippo pathway promotes YAP1 phosphorylation, which is degraded upon binding to the cytoplasmic 14-3-3 protein and is unable to enter the nucleus, losing transcriptional activity [39]. Furthermore, we discovered that the expression of pYAP1 and pLATS1 increased after the knockdown of COPB2 by Western blot.

There are several limitations to this study that warrant further discussion. First, all patients in our study are from Asia; therefore, the results obtained need to be validated in other populations and larger cohorts. Second, the detailed mechanism by which COPB2 regulates chemotherapy sensitivity through modulation of YAP1 needs to be further investigated.

## Conclusion

In conclusion, increased COPB2/YAP1 expression is an independent risk factor in HCC patients and this is correlated with patient sensitivity to drugs. Therefore, COPB2/YAP1 inhibition may be a promising new approach for treating HCC.

## Data Availability

The data that support the findings of this study are available from the authors upon reasonable request.
